# Genomic organization of eukaryotic tRNAs

**DOI:** 10.1186/1471-2164-11-270

**Published:** 2010-04-28

**Authors:** Clara Bermudez-Santana, Camille Stephan-Otto Attolini, Toralf Kirsten, Jan Engelhardt, Sonja J Prohaska, Stephan Steigele, Peter F Stadler

**Affiliations:** 1 Bioinformatics Group, Department of Computer Science and Interdisciplinary Center for Bioinformatics, University of Leipzig, Härtelstraße 16-18, D-04107, Leipzig, Germany; 2 Department of Biology, Universidad Nacional de Colombia. Carrera45 # 26-85 - Edificio Uriel Gutiérrez, Bogotá D.C., Colombia; 3 Genedata AG Maulbeerstrasse 46 CH 4016 Basel, Switzerland; 4 Biostatistics and Bioinformatics unit, Institute for Research in Biomedicine (IRB Barcelona), Barcelona, Spain; 5 Max Planck Institute for Mathematics in the Sciences, Inselstraß 22 D-04103 Leipzig, Germany; 6 Fraunhofer Institute for Cell Therapy and Immunology, Perlickstraße 1, D-04103 Leipzig, Germany; 7 Santa Fe Institute, 1399 Hyde Park Rd, Santa Fe, NM 87501, USA; 8 Institute for Theoretical Chemistry, University of Vienna, Währingerstraße 17, A-1090 Wien, Austria

## Abstract

**Background:**

Surprisingly little is known about the organization and distribution of tRNA genes and tRNA-related sequences on a genome-wide scale. While tRNA gene complements are usually reported in passing as part of genome annotation efforts, and peculiar features such as the tandem arrangements of tRNA gene in *Entamoeba histolytica *have been described in some detail, systematic comparative studies are rare and mostly restricted to bacteria. We therefore set out to survey the genomic arrangement of tRNA genes and pseudogenes in a wide range of eukaryotes to identify common patterns and taxon-specific peculiarities.

**Results:**

In line with previous reports, we find that tRNA complements evolve rapidly and tRNA gene and pseudogene locations are subject to rapid turnover. At phylum level, the distributions of the number of tRNA genes and pseudogenes numbers are very broad, with standard deviations on the order of the mean. Even among closely related species we observe dramatic changes in local organization. For instance, 65% and 87% of the tRNA genes and pseudogenes are located in genomic clusters in zebrafish and stickleback, resp., while such arrangements are relatively rare in the other three sequenced teleost fish genomes. Among basal metazoa, *Trichoplax adhaerens *has hardly any duplicated tRNA gene, while the sea anemone *Nematostella vectensis *boasts more than 17000 tRNA genes and pseudogenes. Dramatic variations are observed even within the eutherian mammals. Higher primates, for instance, have 616 ± 120 tRNA genes and pseudogenes of which 17% to 36% are arranged in clusters, while the genome of the bushbaby *Otolemur garnetti *has 45225 tRNA genes and pseudogenes of which only 5.6% appear in clusters. In contrast, the distribution is surprisingly uniform across plant genomes. Consistent with this variability, syntenic conservation of tRNA genes and pseudogenes is also poor in general, with turn-over rates comparable to those of unconstrained sequence elements. Despite this large variation in abundance in Eukarya we observe a significant correlation between the number of tRNA genes, tRNA pseudogenes, and genome size.

**Conclusions:**

The genomic organization of tRNA genes and pseudogenes shows complex lineage-specific patterns characterized by an extensive variability that is in striking contrast to the extreme levels of sequence-conservation of the tRNAs themselves. The comprehensive analysis of the genomic organization of tRNA genes and pseudogenes in Eukarya provides a basis for further studies into the interplay of tRNA gene arrangements and genome organization in general.

## Background

Transfer RNAs (tRNAs) are among the most ancient genes. They can be traced back to the putative RNA World [1] before the separation of the three Domains of Life. There is clear evidence, furthermore, that all tRNA gene are homologs, deriving from an ancestral " proto-tRNA" [2], which in turn may have emerged from even smaller components, see e.g. [3-7].

Besides their primary ancestral function in translation, tRNAs appear to have acquired several additional modes of employment throughout evolution. Several recent studies, for instance, reported tRNA-derived small RNAs in different Eukaryotic clades [8-12], which at least in part appear to be utilized in the RNAi pathway. Furthermore, tRNA genes are a prolific source of repetitive elements (SINEs) [13], and of tRNA-derived small RNAs such as the small brain-specific non-messenger RNA BC1 RNA [14,15] and other SINE-derived ncRNAs [16].

Multiple copies of functional tRNA genes, the existence of numerous pseudo-genes and tRNA-derived repeats are general characteristics of tRNA evolution throughout Eukarya [17]. In general, tRNA genes appear to evolve rapidly. In *E. coli*, the rate of tRNA gene duplication/deletion events is of the order of one per million years [18], and a recent analysis of schistosome genomes revealed striking differences in the tRNA complement between the close related platyhelminths *S. mansoni *and *S. japonicum *[19].

Although the tRNAs themselves and their sequence and structural evolution has received quite a bit of attention [20-23], much less is known about the genomic organization of tRNA genes. Recent evidence, however, indicates that tRNA genes play a role in eukaryotic genome organization [24] e.g. by acting as barriers that separate chromatin domains. In trypanosomes, for example, tRNA genes mostly appear at the boundaries of transcriptional units and may be involved in the deposition of special nucleosome variants in these regions [25]. Furthermore, there is a link between tRNA loci, in particular clusters of tRNA genes, and chromosomal instability [26-30]. A recent study showed that tRNA genes may act as barriers to DNA replication fork progression [24], providing a possible mechanism for the formation of genomic fragile sites. The genomic evolution of tRNA gene thus may be linked to the evolution of genome organization. Nevertheless, reports on clade-specific features, such as the strong increase of tRNA introns in Thermoproteales [31], are rare.

A peculiar feature of tRNA gene organization are tRNA tandem repeats, which so far have been reported only in the protistan parasite *Entamoeba histolytica *[32,33]. MicroRNAs derived from a precursor in which an imperfectly matched inverted repeat forms a partly double-stranded region, as observed in *Chlamydomonas *[34,35], furthermore, suggests that head-to-head or tail-to-tail arrangements of tRNA gene might form an evolutionary source of small RNAs.

In this contribution, we survey the genomic distribution of tRNA genes and pseudogenes throughout the Eukarya and provide a comprehensive comparative view of the eukaryotic tRNA genomics. Our study makes use of the near-perfect sensitivity and specificity of tRNAscan-SE[36], which reliably determines the complete tRNA complement of eukaryotic genomes.

## Results and Discussion

### Numbers of tDNAs

For each of the 74 genomes included in our survey we collected summary statistics on the number of tRNA gene and tRNA pseudogenes as well as on their genomic clusters. To simplify the language, we will use the term " tDNA" to refer to both tRNA genes and tRNA pseudogenes, while " tRNA gene" will be reserved to loci with probably intact tRNA sequences. In practise, we use tRNAscan-SE to distinguish between tRNA genes and tRNA pseudogenes (see Methods for details).

**Figure 1:**
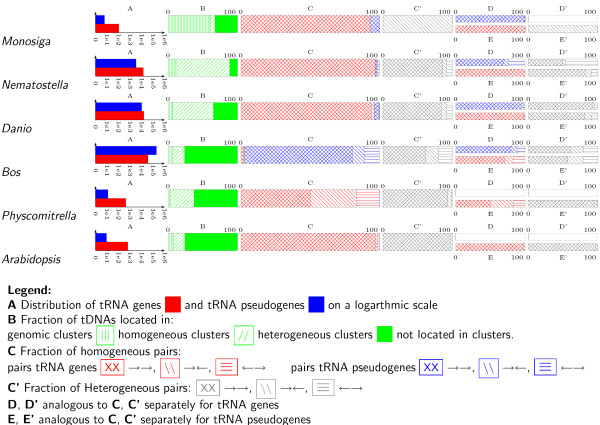
**Summary of tRNA gene and tDNA statistics**.

We define two adjacent tRNA gene or tDNAs as " clustered" if their distance is less than 1000 nucleotides. This threshold is motivated by a statistical analysis of the distances between adjacent tDNA loci, which shows that at this distance we have to expect very few or no tDNA pairs in the genomes under investigation (see Methods for details). We then distinguish between *homogeneous clusters*, consisting of tDNA with the same isoacceptor family (i.e., coding for the same aminoacid), and *heterogeneous clusters*. Within clusters, we separately consider the three relative orientations → →, ← →, and → ←. Data have been analyzed for putatively functional tRNA gene (as classified by tRNAscan-SE), and for all tDNAs. Fig. 1 shows a sample of a graphical representation of the survey results. The full figure comprising all 74 genomes is provided as Additional File 1. Complete lists of tDNAs in gff format can be found at the website [37].

Despite an overall correlation with genome size, there does not seem to be a general trend in the number of tRNA genes. Although some mammals, for instance, exhibit tens or even hundreds of thousands of tDNA copy numbers, other mammalian genomes harbour only a few hundred copies. For instance, old world monkeys and great apes have about 616 ± 120 tDNAs, while the related bushbaby (*Otolemur garnetti*) exhibits 45225 tDNAs. The highest counts are reached for the cow and rat genomes with more than 100000 tDNAs. For the 12 sequenced Drosophila species, we find 320 ± 73 tDNAs. *Trichoplax adhaerans*, one of the most basal animals has no more than 50 tRNA genes, while the cnidaria *Nematostella vectensis *has more than 17000. Within teleosts, tDNAs range from about 700 in Tetraodontiformes to 20000 in zebrafish. Variations by about an order of magnitude are also common in other major clades. *Naegleria gruberi*, for example has 924 tDNAs, while Kinetoplastids (*Leishmania *and *Trypanosoma *have only 91 and 65 copies). Surprisingly, the variation is very small in the " green lineage". Spermatophyta show little variation with 706 ± 96 loci, the basal land plants *Physcomitrella patens *(432 tDNAs) and *Selaginella moellendorffii *(1290 tDNAs) and even the unicellular algae *Volvox carteri *(1051 tDNAs) and *Chlamydomonas reinhardtii *(336 tDNAs) have similar numbers.

**Figure 2:**
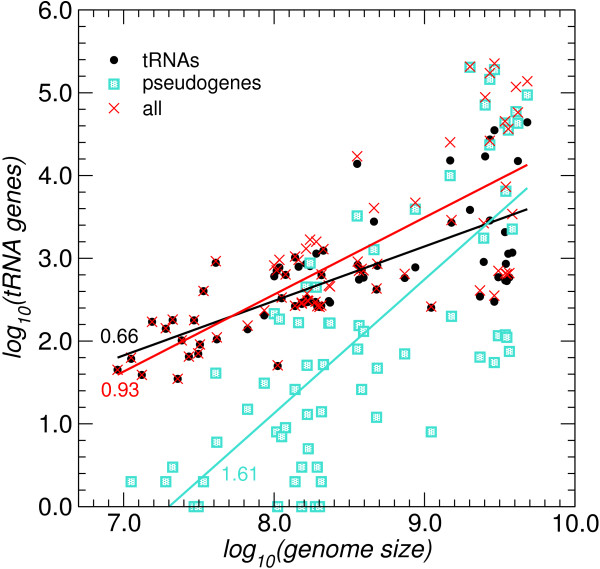
**Correlation of the number of tDNAs with genome size**. The slopes of the three regressions are significantly different: Intact tRNA gene (•, *α *= 0.658 ± 0.076), tRNA pseudogenes (□, *α *= 1.615 ± 0.181), total number of tDNAs (×, *α *= 0.930 ± 0.096).

Despite the often large variation among even closely related lineages, we observe the expected correlation between the number of tDNAs with genome size, Fig. 2. The correlation is significant, with correlation coefficient *ρ *∈ (0.71...0.76), but subject to a high level of variation reflecting large differences in the evolutionary history of different lineages. While the total number of tDNAs scales approximately linearly with genome size, *α *= 0.93 ± 0.10, the growth in the number of intact, probably functional tRNA genes is much slower, consistent with *N*^2/3^. The number of tRNA pseudogenes, on the other hand, grows faster than linearly, ~*N*^1.61 ± 0.18^. The reasons for this difference in scaling remains unclear. One may speculate that selective forces maintain only a limited number of functional tDNA copies causing the sub-linear growth of intact tRNA genes with genome size, while the duplication/deletion mechanism acts towards a uniform coverage of the genome with a rate that is to a first approximation constant throughout eukaryotic genome, accounting for the linear growth of the total number of tDNAs.

Several selective forces could act on the tRNA genes and/or all tDNA loci to cap their number. The bias towards small deletions over insertions observed in [38] is one potential candidate that is independent of special properties of tRNAs. Variations in codon usage might provide another selection-based explanation for the variation of tDNA copy numbers. In eubacteria, a correlation between tRNA abundance, tRNA gene copy number, and codon usage is well established [39]. Whether codon bias causes tDNA copy number variation or *vice versa *remains topic of an intense debate, however. A mechanistic explanation describing the coevolution of codon usage with tRNA gene content is given in [40]. It remains unclear to what extent the correlation of tRNA copy numbers and codon usage carries over to eukaryotic genomes. A detailed investigation in *Schistosoma mansoni *and *Schistosoma japonicum *finds no correlation between tRNA gene numbers and codon usage, while a statistically significant but still very weak correlation is observed in *Schmidtea mediterranea *[19]. In *Nasonia*, the correlation of codon usage and the copy numbers of tRNA genes appears to be restricted to highly expressed genes. The strength of this correlation decreases with GC-content in plant genomes [41].

In any case, codon usage cannot be employed to explain the observed differences in tDNA copy numbers that span several orders of magnitude. These huge fluctuations, which are observed both within some lineages and between closely related lineages, argues against a mechanism that relies on selection on the tRNAs. Instead, the more than linear scaling of tRNA pseudogenes with genome size suggests a faster tDNA turnover in larger genomes - after all, pseudogenes and gene relics are steps in the evolutionary degradation of genes.

### tDNA clusters

In order to investigate the propensity for the formation of tDNA clusters, we consider the cumulative distribution of consecutive tDNA pairs as a function of their genomic distance. Based on a statistical evaluation of the distances between adjacent tDNAs (see Methods), we define two tDNAs to be clustered in the genome if they are located within 1000 nt.

Not surprisingly, in species with small tDNA copy number, clusters typically are rare. In *Trichoplax adherens*, for instance, all tDNAs are isolated. There is no clear-cut relation between tDNA copy number and clustering, however. In *Nematostella vectensis *89% of the tDNAs appear in clusters. In mammals, which have even larger tDNA copy numbers, less than a quarter of the tDNAs appear in clusters. Again, there do not appear to be any large-scale phylogenetic regularities. In teleost fishes, for example, the stickleback *Gasterosteus aculeatus *has 87% clustered tDNAs, in zebrafish this number reaches 65%. On the other hand, pufferfishes and medaka (Oryzias latipes) have predominantly isolated tDNAs. Similarly, large variation appears in other clades, see Fig. 1 and Additional File 2. Higher primates have 17% to 36% of their tDNAs in clusters, with the exception of the bushbaby *Otolemur garnetii*, with only 5.6% of its 45225 tDNAs located in clusters. In plants there are also no clear regularities. The fraction of clustered tDNAs stays below 25% in Spermatophyta, while the chlorophyceae *Volvox carteri *and *Chlamydomonas reinhardtii *have 41% and 56% of their tDNAs localized in genomic clusters.

**Figure 3:**
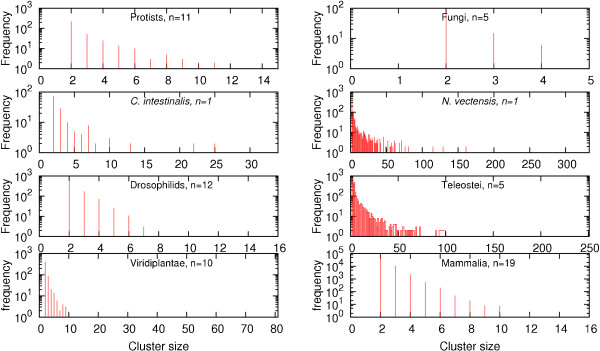
**Distribution of tDNA clusters sizes for several lineages for which multiple sequenced genomes are available as well as some examples of individual genomes**. Most tDNA clusters are small, and the frequency of long clusters rapidly decreases.

Most tDNA clusters are small, containing only a few co-localized tRNA genes. Typically, the frequency of larger clusters quickly decreases, at least approximately following an exponential distribution. This is particularly obvious in the case of mammals and drosophilids. In some cases, however, longer clusters are more abundant. Exceptionally large tDNA gene clusters, with fifty and more members, are observed for example in *Nematostella *and in the genomes of teleost fishes, Fig. 3.

**Figure 4:**
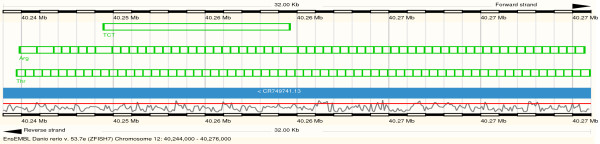
**Example of heterogeneous tDNA cluster consisting of multiple copies of tRNA-Arg(TCT) and tRNA-Thr(AGT or TGT)**. Two tRNA pseudogenes with anti-codon TCT are interspersed.

**Figure 5:**
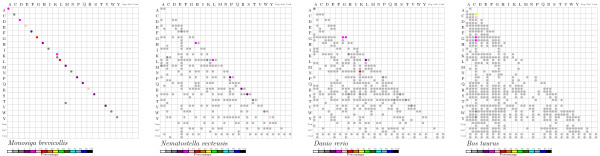
**Relative abundance tRNA isoacceptor families located consecutively within tRNA clusters**. Four data points are shown for each combination of amino acids: Top: pairs in the same reading direction; below: pairs in opposite reading direction. Left: pairs of presumably functional tRNA, right: pairs of tRNA pseudogenes. The last three rows and columns refer to putative Suppressor, SeC, and tRNA pseudogenes of undetermined isoacceptor class, resp.

The internal structure of tDNA clusters also differs widely between lineages. Fig. 1 and Additional Files 2 and 3 summarize the relative abundances of homogeneous and heterogeneous clusters, respectively. More precisely, we record the fraction of adjacent tDNA pairs coding for the same aminoacid. While *Tetrahymena*, *Monosiga*, and the drosophilids exhibit mostly homogeneous pairs, we observe mostly heterogeneous pairs in kinetoplastids, *Nematostella*, clawed frog, and zebrafish, see Fig. 4 for an example. In order to further investigate the structure of heterogeneous clusters we determined how often combinations two isoacceptor families appear in adjacent pairs. These data are conveniently represented in triangular matrices such as those in Fig. 5. Homogeneous clusters populate the main diagonal, whereas heterogeneous pairs are represented by off-diagonal entries. As for other features of the genomic tRNA distribution there are neither strong common patterns among all organisms investigated, nor are there systematic phylogenetic patterns. While *Monosiga*, for example, has almost exclusively homogeneous pairs, other species exhibit a wide variety of heterogeneous pairs. In *Danio*, for instance, K-N, K-S, and R-T are most frequent. In the cow genome, many clusters involve tRNA pseudogenes, which are much less prevalent in the other three examples. In the cow, C-C pseudogenes account for more than 30% of the pairs. A comprehensive collection of co-occurrence tables is provided as Additional File 3. Not surprisingly, there is a general trend towards more complex co-occurrence matrices for species with larger numbers of tDNAs. Most adjacent tDNA pairs in both homogeneous and heterogeneous clusters have parallel orientation. If the arrangements were random, we would expect that 50% of pairs are of this type. In many cases, e.g. *Arabidopsis*, *Selaginella*, *Xenopus*, or *Danio*, nearly all pairs are in parallel. Among the anti-parallel pairs, some species have a strong bias for either head-to-head (e.g. primates, and *Cryptococcus*) or tail-to-tail arrangements (*Oryza *and *Caenorhabiditis*). Even within primates, the ratio of head-to-head and tail-to-tail pairs varies considerably.

In most species with very large tDNA copy numbers we can expect some tDNA clusters to appear by chance. We tested this by randomizing the tDNA locations (see Methods for details). The results for eutherian mammals are compiled in Tab. 1, a full list of random pair configuration is given in Additional File 2. In most genomes, there are significantly more tDNA pairs than expected, suggesting a mode of tDNA evolution of favours the formation of local clusters. Local DNA duplications, also underlying the copy number variations within many populations (see e.g. [42,43] and the references therein), are of course the prime suspects.

**Table 1:** Comparison of observed and expected number of tRNA pairs.

Species	Observed	Expected	p-value
*B. taurus*	28452	22790	0

*C. familiaris*	4858	4271	0

*D. novemcinctus*	7918	11498	1

*M. domestica*	7402	914	0

*E. telfairi*	49	9.35	0

*E. caballus*	72	4.42	0

*F. catus*	8792	11816	1

*G. gorilla*	40	0.08	0

*H. sapiens*	97	0.27	0

*L. africana*	1645	3553	1

*M. mulata*	168	0.23	0

*M. murinus*	42	0.06	0

*M. musculus*	1001	425	0

*O. anatinus*	27015	25008	0

*O. lemur*	1364	1285	0

*P. troglodytes*	78	0.25	0

*P. pygmaeus*	83	0.28	0

*O. cuniculus*	118	37.15	0

*R. rattus*	28198	16148	0

The expectation values are computed by placing the tRNAs at uniformly random position in the genome. Empirical *p *values are computed from 50 to 1000 replicates.

We observe significant under-representations of tDNA pairs only in a few species with very high tDNA counts: *Dasypus novemcinctus*, *Felis catus*, and *Loxodonta africana*. At present, we have no biological explanation for this observation.

Clusters of tDNAs have been implicated in interfering with the DNA replication forks [26]. The tDNA clusters might thus be instrumental in orchestrating the timing of DNA replication. On the other hand, replication fork pause sites are associated with genomic instability [27-30] and hence may contribute to the rapid evolution of these tDNA clusters. Furthermore, retrotransposable elements tend to select tRNA genes as chromosomal integration sites [44], appearently in order to avoid gene disruptions upon retrotransposition. A recent comparison of yeast genomes associated genomic rearrengements, losses, and additions with tRNA genes [45]. Taken together, tDNA clusters thus appear as highly dynamic unstable genomic regions.

### Synteny

Transfer RNAs have been reported to behave similar to repetitive elements as far as their genomic mobility is concerned. They appear to evolve via a rapid duplication-deletion mechanism that ensures that copies of tRNA genes within a genome are usually more similar to each other than tRNA gene of different species [18,46]. In *E. coli*, for example, the rate of tRNA gene duplication/deletion events has been estimated to be about one event every 1.5 million years [18]. We are not aware of (semi)-quantitative estimates from eukaryotes. Our analysis is consistent with this mechanism (see below).

Since tRNA genes with the same anticodon are typically nearly identical, the only way to estimate rates of tRNA gene turnover is to determine, for each tRNA-bearing locus, whether tDNAs can be found in a syntenic locations in evolutionarily related species. We have determined such data here for eight selected species, including six mammals, namely the Catarrihni *Homo sapiens*, *Pan troglodytes*, *Pongo pygmaeus*, and *Macaca mulatta*, the rodent *Mus musculus*, and the Marsupialia *Monodelphis domestica*. The data set includes also more distant vertebrates *Gallus gallus *and *Xenopus tropicalis *to investigate whether there are tDNAs with very stable genomic locations.

Tab. 2 shows the results for the one- and two-side linkage analysis (see Section Methods). The number of related synteny regions based on the single-side linkage analysis is significantly higher than the region number created by the two-side linkage analysis. Since the latter analysis approach is more restrictive, the results between both analysis approaches also differ. While synteny regions in related species are mostly assigned by the single-side linkage analysis, the results of the two-side linkage analysis are more differentiated. Therefore, we discuss only the results of two-side linkage analysis in the following.

**Table 2:** Quantity structure of linkage analysis results in vertebrates

		Homo Sapiens		Pan Troglodytes		Pongo Pygmaeus		Macaca Mulatta		Mus Musculus		Monodelphis Domestica		Gallus Gallus		Xenopus Tropicalis	
Homo Sapiens	493			22,312		20,143		17,154		453,512		167,537		985		6341	
				444	494	438	488	438	387	442	22,206	442	20,398	366	155	383	332
				0.9	0.98	0.89	0.95	0.89	0.86	0.9	0.91	0.9	0.94	0.74	0.93	0.78	0.57

Pan Troglodytes	504	8,641				22,375		17,073		176,153		182,022		1,048		6,201	
		366	394			497	503	498	391	504	22,963	503	20,847	400	160	395	364
		0.73	0.8			0.99	0.98	0.99	0.87	1	0.94	1	0.96	0.79	0.96	0.78	0.62

Pongo Pygmaeus	512	8,673		7,556				16,838		179,360		183,128		1,033		6,585	
		349	368	330	375			494	390	512	22,716	512	20,797	411	158	450	348
		0.68	0.75	0.64	0.74			0.96	0.87	1	0.93	1	0.95	0.8	0.95	0.88	0.59

Macaca Mulatta	450	6,301		5,881		5,488				152,984		152,619		909		5,550	
		309	368	284	363	286	333			393	22,588	393	20,646	332	156	355	347
		0.69	0.75	0.63	0.72	0.64	0.65			0.87	0.93	0.87	0.95	0.74	0.94	0.79	0.59

Mus Musculus	24352	6,212		5,958		6,289		5,151				10,073,201		65,044		106,441	
		2,030	382	2,294	369	2,126	395	2,211	351			24,336	21,809	20,643	166	20,716	422
		0.08	0.77	0.09	0.73	0.09	0.77	0.09	0.78			1	1	0.85	1	0.85	0.72

Monodelphis Domestica	21810	3,395		3,766		3,677		4,017		190,815				67,383		106,233	
		1,750	318	2,071	363	1,846	35	2,123	353	16,634	19,466			19,290	166	19,306	416
		0.08	0.65	0.09	0.72	0.08	0.07	0.1	0.78	0.76	0.8			0.88	1	0.89	0.71

Gallus Gallus	166	44		46		43		42		1,398		1,560				569	
		38	38	38	39	35	38	32	38	132	1,169	130	1276			142	236
		0.23	0.08	0.23	0.08	0.21	0.07	0.19	0.08	0.8	0.05	0.78	0.06			0.86	0.4

Xenopus Tropicalis	586	77		74		79		59		912		802		16			
		24	72	25	69	23	65	26	53	126	708	115	630	16	12		
		0.04	0.15	0.04	0.14	0.04	0.13	0.04	0.12	0.22	0.03	0.2	0.03	0.03	0.07		

The upper right triangle quantifies the single-sided linkage results whereas the lower left triangle represents the number of two-sided linkage analysis results. Each table entry is organized as follows: The top row lists the number of synteny associations; Below, the the sizes of domain and range, i.e., the numbers of tDNAs in the two species, are given. The third line gives the corresponding coverage, i.e., the fraction of syntenically conserved tDNAs in the two species.

Within Catarrhini, tDNA locations are quite well conserved. For instance 80% (394/493) of human tDNA regions are conserved in the chimp, and there are still 63% (284/450) of the rhesus tDNA locations recovered in chimp. Somewhat surprisingly, there is also a large fraction of syntenic loci between mouse and opossum (80% [19,466/24,352] of the mouse loci and 76% [16,634 of 21,810] of the opossum loci). We suspect that the large fraction is confounded by the large overall number of tDNA loci and the rather larger intervals of five flanking genes used to define synteny, which taken together cover a substantial fraction of the genome. A second group of comparisons identified only a small number of syntenically conserved loci. Asymmetric results, which large retention in one direction is observed when the tDNA numbers are dramatically different. This concerns the comparisons between Catharrini, on the one hand, and opossum and mouse on the other hand. Between frog and Catharrini, finally, there is only a small number of syntenically conserved tDNAs.

We also analyzed the tDNA mobility in two invertebrate clades, drosophilids and nematode genus Caenorhabditis. Within these nematodes, we observe a rather high degree of syntenic conservation, ranging from 45% between *C. elegans *and *C. japonica *up to 84% for the most closely related pair *C. remanei *and *C. brenneri*. In general, conservation levels are consistent to the known phylogeny of the *Caenorhabditis *species [47]. For the genus Drosophila with the twelve common representatives, on the other hand, there is much less syntenic conservation. The lowest value is 17% (*D. wilistoni *and *D. persimilis*). The best conserved tDNA arrangements are observed between the two closely related species *D. simulans *and *D. sechellia *with 78%. On average, the percentage of conservation is just around 50% or less. Full data are shown in Tab. 3 for nematodes and in Additional File 4 for Drosophila.

**Table 3:** Syntenic conservation of tDNAs

	tDNA	*C. briggsae*	*C. remanei*	*C. brenneri*	*C. elegans*	*C. japonica*
*C. briggsae*	958	-	0.84 809	0.82 788	0.72 691	0.68 647
*C. remanei*	958	0.74 712	-	0.73 696	0.63 603	0.55 528
*C. brenneri*	1587	0.61 962	0.63 997	-	0.49 783	0.48 763
*C. elegans*	820	0.77 629	0.75 617	0.73 602	-	0.68 558
*C. japonica*	1307	0.46 607	0.48 633	0.49 634	0.45 589	-

The table shows the fraction of tRNA loci between pairs of species. Every field contains the fraction of tDNAs of the species in the column, for which we could find a syntenic position in the row species.

The sequence conservation of syntenically conserved tRNAs is consistent with the duplication/deletion mechanisms. Additional File 5 shows a neighbor-joining tree of the tRNA-Ala sequences of nematodes, which includes also a few additional species that are not part of the genome-wide survey. We find that syntenically conserved tRNAs genes are typically conserved with an identical sequence across species, even though some tRNAs with the same anticodon located elsewhere in the genome show small sequence variations.

**Figure 6:**
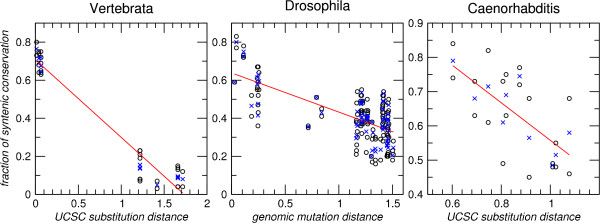
**Correlation of syntenic conservation of tDNA loci with genomic distance**. Estimates for each pairwise comparisons (◦) and averages over the two comparisons for each pair of species (×) are shown. For vertebrates and nematodes distances were extracted from trees provided through the UCSC browser, for Drosophilds, corrected mutation distances were used (see Methods for details). Because of the large number of tDNA loci *Mus musculus *and *Monodelphis domestica *were not used for the correlation. Parameters of the linear regression are compiled in Tab. 4

The fraction of syntenically conserved tDNAs correlates with the divergence of the genomes at sequence level, Fig. 6 and Tab. 4. The correlation is significant even though the data is rather noisy, a fact that can be explained at least in part by the unavoidable artifacts resulting from our approach. Utilizing annotation data directly to determine local synteny is problematic, for instance, near members of very large recently duplicated gene families. In principle, syntenic conservation could be inferred more accurately from genome-wide alignments. Since tDNAs are treated like repetitive elements in the currently available pipelines, this strategy cannot be employed in practice. Nevertheless, the method provides at least a crude estimate of the tDNA turnover rate, indicating the tDNAs are relocated at time-scales only 2-5 times slower than background mutation rate, i.e., at an evolutionary distance of 1 mutation per site, 20% to 60% of the tDNAs have been deleted or relocated in one lineage.

**Table 4:** Parameters of the linear regressions in Fig. 6

Clade	*ρ*	slope
Vertebrates	-0.968	-0.41 ± 0.02
Drosophila	-0.678	-0.21 ± 0.02
Caenorhabditis	-0.638	-0.55 ± 0.16

These values should be regarded as upper bounds of syntenic conservation, i.e., tDNA turnover is probably even faster. For example, the identity of the tDNA (i.e., its anticodon) was not used in the analysis. Despite of the high mobility of tDNAs there are some ancient conserved loci. We further investigated two of the 77 syntenic loci conserved between *Xenopus *and *Human *in which tDNAs with the same anticodon were retained. Manual inspection of the flanking protein coding genes confirmed synteny. Neither locus is syntenically conserved in stickleback, lamprey or lancet, however.

## Conclusions

We have developed a pipeline based on tRNAscan-SE[36] to extract and analyze the locations of tRNA genes and pseudogenes of eukaryotic genomes. In our analysis, we focus not only on the number of tRNA genes, but also on their relative genomic locations, and in particular on the formation of tDNA clusters. Surprisingly, we found no distinctive clade-specific features or large scale trends, with the exception of the rather straightforward observation that the larger metazoan genomes typically tend to harbour large numbers of tDNAs.

In some species, large clusters of tDNAs occur. This effect has first been reported in *Entamoeba histolytica*. The origin of this gene organization in the genus Entamoeba clearly predates the common ancestor of the species investigated to date. Their function of the array-like structure remains unclear [32]. We report here that this phenomenon is not restricted to a particular clade of protists but rather appears independently in many times throughout eukaryotes.

In most eukaryotes, tRNAs are multi-copy genes with little or no distinction between paralogs so that orthology is hard to establish, in particular in the presence of tRNA gene clusters. As a consequence, the evolution of genomic tRNA arrangements is non-trivial to study over larger time-scales. Upper bounds on syntenic conservation can be estimated, however, by considering small sets of flanking protein coding genes for which homology information can be retrieved from existing annotation. We found that tRNAs change their genomic location at time-scales comparable to mutation rates: syntenic conservation fades at roughly the same evolutionary distances as sequence conservation in unconstrained regions.

The absence of large numbers of partially degraded tRNA copies in many of the investigated genomes provides a hint at the mechanisms of tRNA mobility: At least in part the relocation events appear to be linked to chromosomal rearrangements rather than mere duplication-deletion of the tRNA genes themselves. The latter mechanism, which appears to be prevalent e.g. in mitochondrial genomes [48], certainly also plays a role, since tRNA pseudogenes are readily observed in many species, as do tRNA retrogenes [49]. A link between tRNA loci, and in particular tRNA clusters, and chromosomal instability has been pointed out repeatedly in the literature, showing that tRNA genes can interfere with the replication forks [26-30]. The data collected here provide a basis to investigate this connection more systematically in the future.

Overall, the tRNA complement of Eukaryotes is highly dynamic part of the genomes whose organization evolves rapidly and in a highly lineage specific manner - a behavior that is in striking contrast to the extreme conservation of sequence and function of the tRNAs themselves.

## Methods

### Sequence data

We retrieved 74 eukaryotic genome mainly from the following public resources: NCBI, Ensemble Genome Browser and Joint Genome Institute. For a detailed list of the individual genome assemblies we refer to the Additional File 6.

### tRNA detection

Detection of tRNAs was performed by using tRNAscan-SE v.1.23 (April 2002) with default parameters, i.e., the TRNA2.cm covariance with strict filter parameter 32.1 was used to screen each genome for tRNAs and tRNA pseudogenes. All analyses were performed using both the set of all intact, putatively functional tRNAs identified by tRNAscan-SE and using all tDNA loci, i.e., the union of tRNA genes and tRNA pseudogenes.

The distinction of tRNA genes and pseudogenes necessarily relies of a set of heuristics implemented in tRNAscan-SE. These are well-founded in what is known about functional tRNA genes [50-55]. Processing and recognition of specific tRNAs imposes stringent constraints on the sequence (and secondary structure) of tRNAs; several nucleotides of mature tRNAs need to chemically modified in most species, imposing further constraints on the primary sequence. tRNAscan-SE's consensus models implement these contraints with reasonable accuracy but by no means perfectly. In the absence of detailed experimental information on the expression and the functionality of a particular tDNA it is of course impossible to distinguish between tRNA genes and tRNA pseudogenes with absolute certainty. For the statistical evaluation of genome-wide comparison reported here, however, the accuracy of tRNAscan-SE appears to be sufficient [21,36,56]. There are, however, several sources for errors, in particular in the presence of RNA editing e.g. in the mitochondrial tRNAs of many plants and the protist [57-60]. Such organellar data are not considered in this contribution, however.

tRNA-geo **pipeline **The tRNA-geo pipeline is a Perl program that parses tRNAscan-SE output and produces summary information as well as overview graphics such as those shown in Figs. 1 and 5. First tDNA locations are sorted in consecutive order along each input sequence, distances are measures (see below for exact definitions), tDNA pairs and tDNA clusters are identified, summary statistics are computed. Graphics are produced using PSTricks macros and LaTeX.

Every tDNA is represented by a quadruple *P *= (*a*, *b*, *o*, *t*), where *a < b *are the start and end positions within each input sequence (chromosome, scaffold, or contig), and *o *∈ {+, -} is the orientation of the tRNAs. We say that two tDNAs are of the same type *t *if they belong the same isoacceptor family, i.e., if they code for the same aminoacid. The tRNA loci are ordered such that *P*_*i *_≺ *P*_*j *_if and only if *a*_*i *_<*a*_*j*_. The distance between two consecutive loci *P*_*i *_and *P*_*i*+1 _is defined as *δ*_*i *_= *a*_*i*+1_-*b*_*i*_.

**Figure 7:**
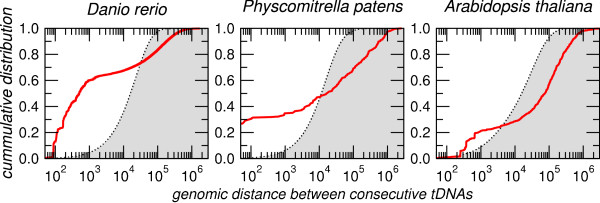
**Cumulative distribution of tDNA pairs distances**. Measured data are shown in red, random expectation from randomly placed tDNAs are shown as gray background. At a distance of 1000 nt the vast majority of clusters cannot be explained by the random background.

A cluster **C **is a maximal sub-sequence of loci **C **= (*P*_*i*_, *P*_*i*+1_, ..., *P*_*j*_) such that *δ*_*k *_< 1000 for *i *≤ *k *<*j*. The cut-off of 1000 was chosen because the overwhelming majority of consecutive tDNA pairs in the random control have larger distances while a large fraction of the tDNA pairs in the real data have smaller distances than this cut-off value, see Fig. 7 here.

A cluster is called homogeneous if all its tDNAs are of the same type *t*_*k*_; otherwise, it is called heterogeneous. A sub-sequence consisting of two consecutives loci located within a cluster **C **is called a pair. The pair (*P*_*i*_, *P*_*i*+1_) is homogeneous if *t*_*i *_= *t*_*i*+1 _and heterogeneous otherwise. A pair has parallel orientation if *o*_*i *_= *o*_*i*+1_. For anti-parallel pairs, *o*_*i *_= *o*_*i*+1_, we distinguish head-to-head *o*_*i *_= + and *o*_*i*+1 _= - (← →), and tail-to-tail *o*_*i *_= - and *o*_*i*+1 _= + (→ ←) orientations.

In order to test whether the observed proportion of homogenous and inhomogeneous pairs depends strongly on whether tRNA pseudogenes are included in the analysis, we used Fisher's exact test. Differences in proportion are significant only in a few species, typically those with very large number of tDNAs (see Additional File 7) suggesting that pseudogenization and degradation of tRNA genes is to a first approximation independent of the mutual positioning of tDNAs.

### Simulations

In order to investigate the statistical significance of the tDNA pairs we compare the genomic tDNA organization with randomized configurations. To this end, we remove the collection of tRNA genes and pseudogenes from the genome and re-insert them at positions chosen from a uniform distribution on the remaining sequence. Empirical *p*-values, defined as(1)

where *y*(*i*) is the number of clustered tRNAs in replicate *i*, *x *is the number of clustered tRNAs in the genome, are determined from *N *= 50 to *N *= 1000 random replicates. For large (insignificant) *p*-values, simulations were terminated at fewer replicates to save computer time.

**Statistical tests **were performed using the R statistics environment [61]. In particular, Fisher's exact test [62] with 2 × 2 contingency tables was used to determine whether different filtering procedures influenced the proportion of homogeneous versus heterogeneous tDNA pairs.

### Synteny

To analyze the synteny between species, we utilized two different pipelines depending on available genomic data and their interrelations in public data sources. The BioFuice [63] integration platform is used to analyze the synteny in eight different vertebrate species *Homo sapiens*, *Pan troglodytes*, *Pongo pygmaeus*, *Macaca mulatta*, *Mus musculus*, *Monodelphis domestica*, *Gallus gallus*, and *Xenopus tropicalis*. The analysis runs in several steps. Firstly, the Ensembl data source (version 53) is utilized to create the genomic mappings between the tRNAs and/or tRNA pseudogenes and at most five consecutive protein-coding flanking genes in both directions, up- and downstream. The number 5 was chosen pragmatically as a trade-off between the need to evaluate local information and the unavoidable incompleteness of genome annotations, whence homologs of many genes are missing in individual genomes. These genomic mappings are chromosome- and strand-specific, i.e., the resulting genes are located on the same chromosome and strand as the input tDNAs. Next, the resulting genes are associated to protein-coding genes of other mammalian species using the homologous data available in Ensembl Compara (version 53). These homology relationships between genes in different species are then filtered to focus on those genes flanking tRNAs. Finally, tDNAs of different mammals can be associated based on the genomic mappings to their flanking genes (gene-tRNA) and the homology relations between those (gene-gene). We consider two alternatives for creating such tDNA relationships:

1. Two tDNAs are associated by the *single-sided linkage *relation if there is at least one homology relationship between their pre-selected flanking genes. Here we do not require that the homologous genes have the same relative orientation or relative location w.r.t. to the tDNAs.

2. Two tDNAs are associated by the *two-sided linkage *relation if there is at least one pair of homologous genes in both the up-stream and the down-stream region. Again, relative orientations are not taken into account. The tDNAs need to be located between the two homologous gene-pairs, however.

The Single-sided linkage relation turns out to be not very informative because many-to-many homology relations for large gene families and the relatively large regions used to define the synteny relation severely limit the sensitivity. We therefore limit a details discussion to the two-sided linkage relation.

For invertebrate genomes, synteny information was extracted directly from genome annotation using a custom-made pipeline based on Perl and awk scripts. For the nematodes *C. elegans*, *C. briggsae*, *C. japonica*, *C. brenneri*, *C. remanei *we considered a region of 40.000 nt up- and downstream of the tRNA loci. A pair of tDNAs was defined as syntenic if we could found in this range at least two orthologous proteins between them. The flanking proteins were taken from the genome annotation gff-files from Wormbase WS204. A list of orthologous proteins was computed using *OrthoMCL *[64] to determine if two proteins are ortholog. Tab. 3 summarizes the prevalence of tRNA synteny within the genus Caenorhabditis. The tDNAs in the genus Drosophila were analyzed in the same way. The flanking proteins were take from Flybase (release FB2009_09). Since a sufficiently complete orthology annotation was not readily available, we used ProteinOrtho [65] for this purpose. The results are compiled in Additional File 4.

The fraction of syntenically conserved tDNAs was compared to the evolutionary distances for each pair of genomes in the three data sets described above. The evolutionary distance for the Vertebrates and Nematodes is gathered by the tree model underlying the UCSC 28-way alignments [66]. For the genus Drosophila the evolutionary distances are genomic mutation distances computed from 4-fold degenerated sites in all coding regions corrected for base composition as in [67].

## Authors' contributions

CBS and PFS designed the study, CBS, CSOA, TK, and JE implemented analysis tools and analyzed the data, all authors contributed to the interpretation of the results, CBS and PFS drafted the manuscript, and all authors contributed to, read, and approved the final manuscript.

## Supplementary Material

Additional file 1 **Genomic Distribution of tDNAs**. Comprehensive overview of the genomic distribution of tRNA genes and tRNA pseudogenes as described in Fig. 1. Click here for file

Additional file 2 **Count of tDNAs and tDNA pair configurations**. List of total tDNA predictions including *P*-values and counts of tDNA pair configurations from the empirical data and simulations. Click here for file

Additional file 3 **Co-occurrences of tDNAs**. Comprehensive summary of co-occurrence data for tDNAs as described in Fig. 5. Click here for file

Additional file 4 **Syntenic conservation of Drosophila tDNAs**. List of percentage distribution of tDNA synteny in the genus *Drosophila*. Click here for file

Additional file 5 **Sequence conservation of nematode tDNA-Ala**. Neighbor-joining tree showing that tDNAs usually have identical sequences when they are syntenically conserved, while tRNAs with the same anticodon can exhibit small sequence variations within each species. Click here for file

Additional file 6 **Genome versions used in this survey**. List of the genome versions and website from which they were downloaded. Click here for file

Additional file 7 **Proportions of tDNA pairs**. Summary of the Fisher Test statistic for comparing proportion of pairs configurations. Click here for file
