# Supplementary figures and images for: Genomic organization of eukaryotic tRNAs
Source: BMC Genomics. 2010 Apr 28;11:270. doi: 10.1186/1471-2164-11-270 (PMC2888827; doi:10.1186/1471-2164-11-270)

# Genomic distribution of tRNA genes and tRNA<sup>A</sup>

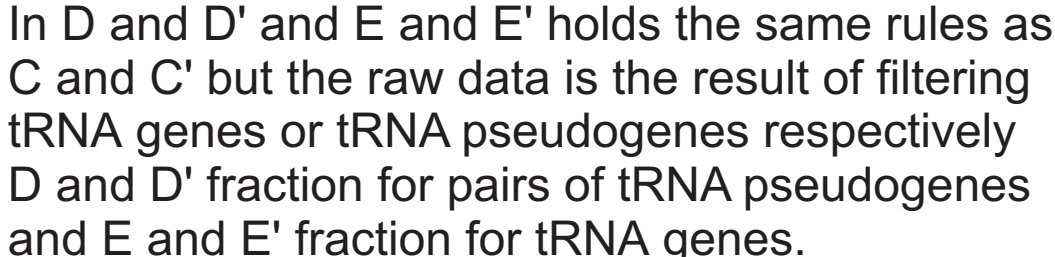

Supplement: Additional file 1 — Genomic Distribution of tDNAs. Comprehensive overview of the genomic distribution of tRNA genes and tRNA pseudogenes as described in Fig. 1. [file 1471-2164-11-270-S1.PDF]
