# Supplementary material for: Genomic organization of eukaryotic tRNAs
Source: BMC Genomics. 2010 Apr 28;11:270. doi: 10.1186/1471-2164-11-270 (PMC2888827; doi:10.1186/1471-2164-11-270)
Supplement: Additional file 2 — Count of tDNAs and tDNA pair configurations. List of total tDNA predictions including P-values and counts of tDNA pair configurations from the empirical data and simulations. [file 1471-2164-11-270-S2.PDF]

Summary of counts of tRNAscan-SE detections, tRNAs, tRNAs pseudogenes, Pairs and Counting of Pairs Configurations

Specie	Total	Pseu	tRNA	%C	%noCl	GS	O.	R.	P	Ho++	Ho+-	Ho-+	He++	He+-	He-+	R Ho++	R Ho+-	R Ho-+	R He++	R He+-	R He-+
Trypanosoma brucei	65	0	65	84.62	15.38	2.71E+007	37	0.22	0	0	0	2	17	9	9	0.01	0	0	0.11	0.05	0.05
Leishmania infantum	91	0	91	86.81	13.19	3.22E+007	58	0.36	0	1	0	1	28	12	16	0.01	0	0	0.17	0.09	0.08
Naegleria gruberi	924	41	883	16.13	83.87	4.10E+007	77	39.35	0	8	4	0	45	13	7	1.02	0.51	0.51	18.64	9.34	9.33
Giardia lamblia	63	2	61	6.35	93.65	1.12E+007	2	0.97	0	0	0	0	1	1	0	0.02	0.01	0.01	0.46	0.23	0.23
Plasmodium falciparum	35	0	35	51.43	48.57	2.29E+007	9	0.09	0	0	0	0	1	7	1	0	0	0	0.04	0.02	0.02
Cryptosporidium parvum	45	0	45	13.33	86.67	9.10E+006	3	0.4	0	1	0	1	0	0	1	0.01	0	0	0.19	0.1	0.09
Tetrahymena thermophila	714	8	706	65.27	34.73	1.04E+008	341	12.53	0	311	3	7	17	3	0	0.35	0.14	0.15	5.99	2.95	2.95
Phytophthora sojae	235	31	204	43.4	56.6	8.60E+007	67	0.9	0	56	2	0	7	1	1	0.03	0.01	0.01	0.42	0.21	0.21
Phytophthora ramorum	154	15	139	42.21	57.79	6.67E+007	38	0.49	0	34	0	0	4	0	0	0.01	0.01	0.01	0.23	0.11	0.12
Thalassiosira pseudonana	71	1	70	25.35	74.65	3.13E+007	9	0.29	0	2	0	0	7	0	0	0.01	0	0	0.14	0.07	0.07
Dictyostelium discoideum	403	2	401	9.43	90.57	3.39E+007	19	8.23	0	4	1	1	9	4	0	0.23	0.12	0.12	3.88	1.95	1.94
Batrachochytrium dendrobatidis	102	0	102	1.96	98.04	2.43E+007	1	0.8	0	0	0	1	0	0	0	0.02	0.01	0.01	0.39	0.19	0.19
Sporobolomyces roseus	182	3	179	52.2	47.8	2.12E+007	56	3.13	0	2	3	1	29	15	6	0.08	0.04	0.04	1.48	0.74	0.74
Cryptococcus neoformans	143	2	141	9.79	90.21	1.91E+007	7	2.55	0	3	3	0	1	0	0	0.07	0.04	0.04	1.22	0.6	0.6
Aspergillus fumigatus	179	1	178	35.2	64.8	2.94E+007	38	1.66	0	19	3	11	4	1	0	0.04	0.02	0.02	0.78	0.4	0.39
Pichia stipitis	171	0	171	33.33	66.67	1.54E+007	30	3.15	0	4	0	1	21	2	2	0.08	0.04	0.04	1.52	0.73	0.73
Monosiga brevicollis	111	6	105	67.57	32.43	4.16E+007	49	0.65	0	47	0	0	0	2	0	0.02	0.01	0.01	0.3	0.16	0.16
Trichoplax adhaerans	51	1	50	0	100	1.06E+008	0	0.03	1	0	0	0	0	0	0	0	0	0	0.02	0.01	0.01
Nematostella vectensis	17076	3249	13827	88.73	11.27	3.57E+008	14143	1124.68	0	8592	40	144	4590	335	442	38.64	14.11	14.13	537.92	259.99	259.88
Lottia gigantea	912	80	832	90.68	9.32	3.60E+008	629	3.34	0	102	0	2	318	94	113	0.09	0.05	0.05	1.58	0.79	0.79
Drosophila simulans	267	2	265	52.43	47.57	1.38E+008	83	0.7	0	44	5	6	12	6	10	0.02	0.01	0.01	0.34	0.17	0.17
Drosophila sechellia	309	13	296	60.52	39.48	1.67E+008	113	0.73	0	53	14	11	16	7	12	0.02	0.01	0.01	0.35	0.17	0.17
Drosophila melanogaster	304	5	299	65.46	34.54	1.69E+008	124	0.93	0	59	16	16	15	7	11	0.02	0.01	0.01	0.44	0.22	0.22
Drosophila yakuba	375	51	324	55.2	44.8	1.66E+008	129	1.15	0	61	17	15	15	8	13	0.03	0.02	0.02	0.54	0.27	0.27
Drosophila erecta	284	3	281	62.68	37.32	1.53E+008	110	0.74	0	49	15	14	14	6	12	0.02	0.01	0.01	0.36	0.17	0.17
Drosophila ananassae	469	165	304	50.75	49.25	2.31E+008	141	1.2	0	64	13	12	34	8	10	0.06	0.03	0.03	0.54	0.27	0.27
Drosophila pseudoobscura	292	1	291	57.53	42.47	1.53E+008	107	0.79	0	54	11	11	12	9	10	0.02	0.01	0.01	0.37	0.19	0.19
Drosophila persimilis	298	1	297	57.38	42.62	1.88E+008	107	0.61	0	51	11	11	12	11	11	0.02	0.01	0.01	0.29	0.15	0.14
Drosophila willistoni	457	165	292	38.95	61.05	2.36E+008	109	1.13	0	50	16	18	9	10	6	0.07	0.04	0.04	0.49	0.25	0.25
Drosophila mojavensis	264	3	261	48.11	51.89	1.94E+008	79	0.49	0	35	11	9	10	8	6	0.01	0.01	0.01	0.23	0.12	0.12
Drosophila virilis	269	2	267	51.67	48.33	2.06E+008	86	0.46	0	40	12	8	12	9	5	0.01	0.01	0.01	0.22	0.11	0.11
Drosophila grimshawi	259	1	258	47.49	52.51	2.00E+008	76	0.42	0	40	8	4	11	9	4	0.01	0.01	0.01	0.2	0.1	0.1
Caenorhabditis briggsae	958	184	774	30.48	69.52	1.08E+008	172	8.22	0	43	17	45	24	12	31	0.17	0.08	0.08	3.95	1.97	1.97
Caenorhabditis remanei	958	167	791	25.05	74.95	1.45E+008	134	9.86	0	35	9	32	29	8	21	0.2	0.1	0.1	4.73	2.37	2.37

Caenorhabditis brenneri	1587	448	1139	26.4	73.6	1.91E+008	233	16.9	0	66	14	50	61	7	35	0.31	0.15	0.15	8.13	4.08	4.07
Caenorhabditis elegans	820	214	606	40.61	59.39	1.00E+008	195	12.86	0	50	30	47	37	18	13	0.23	0.11	0.11	6.19	3.11	3.11
Caenorhabditis japonica	1307	450	857	20.28	79.72	1.63E+008	151	16.02	0	40	25	32	21	13	20	0.41	0.21	0.21	7.59	3.81	3.8
Ciona intestinalis	1680	875	805	41.01	58.99	1.73E+008	540	17.56	0	275	9	6	161	39	50	0.66	0.32	0.33	8.15	4.06	4.04
Danio rerio	25194	9986	15208	65.29	34.71	1.48E+009	14667	679.69	0	4994	105	25	7994	795	754	26.17	12.77	12.77	313.72	157.12	157.13
Tetraodon nigroviridis	707	154	553	28.15	71.85	3.67E+008	117	1.95	0	60	2	4	38	5	8	0.04	0.02	0.02	0.94	0.47	0.47
Takifugu rubripes	716	131	585	29.33	70.67	3.93E+008	131	1.96	0	82	7	4	31	4	3	0.05	0.02	0.02	0.93	0.46	0.46
Gasterosteus aculeatus	4046	1274	2772	87.3	12.7	4.62E+008	3144	57.18	0	1982	4	1	1078	39	40	3.05	1.32	1.32	25.58	12.97	12.95
Oryzias latipes	4695	3919	776	8.97	91.03	8.69E+008	286	35.41	0	158	8	0	72	25	23	4.38	2.19	2.19	13.32	6.67	6.67
Xenopus tropicalis	2894	199	2695	79.75	20.25	1.51E+009	1768	7.8	0	553	14	23	740	205	233	0.23	0.1	0.1	3.67	1.85	1.85
Ornithorhynchus anatinus	206915	203073	3842	23.68	76.32	2.01E+009	27015	25008.22	0	8227	1484	2396	9446	2090	3372	4951.19	2475.69	2475.35	7552.34	3777.16	3776.48
Monodelphis domestica	36542	35408	1134	40.32	59.68	3.61E+009	7402	914.11	0	99	34	23	7169	36	41	121.01	60.58	60.49	336.05	167.92	168.05
Dasypus novemcinctus	137797	93845	43952	11.1	88.9	4.82E+009	7918	11498.56	1	1741	192	448	3849	607	1081	2447.66	642.51	643.47	5089.8	1337.31	1337.8
Oryctolagus cuniculus	7324	6466	858	2.76	97.24	3.47E+009	118	37.15	0	11	3	2	63	9	30	5.8	2.9	2.9	12.77	6.39	6.39
Mus muscullus	26264	23401	2863	7.28	92.72	2.72E+009	1001	425.51	0	250	78	75	343	122	133	77.96	38.99	38.97	134.86	67.37	67.35
Rattus norvegicus	172474	145265	27209	28.49	71.51	2.72E+009	28198	16148.13	0	3512	929	1022	13927	4303	4505	1689.01	844.74	844.69	6385.04	3191.61	3193.04
Echinops telfairi	3426	2255	1171	2.69	97.31	3.83E+009	49	9.35	0	4	1	1	29	6	8	0.38	0.19	0.19	4.3	2.15	2.15
Canis familiaris	88179	71169	17010	10.58	89.42	2.53E+009	4858	4271.27	0	426	157	178	2332	829	936	339.71	169.5	169.75	1796.13	898.29	897.88
Felis catus	117583	59100	58483	14.22	85.78	4.06E+009	8792	11816.7	1	1604	246	234	4808	886	1014	1435.56	717.16	717.98	4473.48	2237.22	2235.3
Bos taurus	225600	190329	35271	23.62	76.38	2.92E+009	28452	22790.52	0	6151	697	876	12716	3721	4291	2125.85	1063.02	1063.46	9269.66	4634.56	4633.98
Equus caballus	2656	1752	904	4.86	95.14	2.47E+009	72	4.42	0	7	5	2	32	9	17	0.4	0.2	0.2	1.81	0.9	0.9
Loxodonta africana	57804	42827	14977	5.57	94.43	4.18E+009	1645	3553.2	1	204	70	85	751	207	328	387.44	193.65	193.96	1389.3	694.93	693.93
Otolemur garnettii	45225	43155	2070	5.89	94.11	3.43E+009	1364	1285.4	0	531	133	162	314	101	123	394.91	197.63	197.6	247.71	123.82	123.73
Microcebus murinus	354	55	299	21.19	78.81	2.91E+009	42	0.06	0	4	2	0	23	2	11	0	0	0	0.03	0.01	0.01
Macaca mulata	706	116	590	36.26	63.74	3.10E+009	168	0.23	0	67	4	2	70	13	12	0	0	0	0.11	0.06	0.06
Pongo pygmaeus	659	119	540	22.31	77.69	3.44E+009	83	0.28	0	9	5	3	37	14	15	0.01	0	0	0.13	0.07	0.07
Gorilla gorilla	409	64	345	17.11	82.89	2.34E+009	40	0.08	0	3	1	0	23	6	7	0	0	0	0.04	0.02	0.02
Pan troglodytes	643	111	532	21.62	78.38	3.52E+009	78	0.25	0	9	6	1	35	13	14	0.01	0	0	0.12	0.06	0.06
Homo sapiens	663	75	588	25.64	74.36	3.67E+009	97	0.27	0	8	5	2	45	16	21	0.01	0	0	0.13	0.07	0.06
Gallus gallus	261	8	253	36.4	63.6	1.11E+009	66	0.1	0	14	6	2	21	10	13	0	0	0	0.05	0.02	0.02
Ostreococcus lucimarinus	39	0	39	12.82	87.18	1.32E+007	3	0.2	0	2	0	0	0	0	1	0	0	0	0.09	0.05	0.05
Chlamydomonas reinhardtii	336	7	329	56.55	43.45	1.12E+008	136	2.04	0	18	8	9	91	8	2	0.06	0.03	0.03	0.98	0.47	0.47
Volvox carteri	1051	26	1025	41.01	58.99	1.38E+008	249	17.05	0	49	0	5	159	29	7	0.5	0.23	0.23	8.03	4.02	4.03
Physcomitrella patens	432	12	420	37.27	62.73	4.80E+008	85	0.45	0	3	2	1	74	4	1	0.01	0.01	0.01	0.22	0.11	0.1
Selaginella moellendorffii	1290	52	1238	15.35	84.65	2.13E+008	110	8.99	0	47	1	4	37	11	10	0.24	0.12	0.12	4.25	2.13	2.13
Popolus trichocarpa	858	47	811	10.96	89.04	4.86E+008	59	1.79	0	10	2	1	31	10	5	0.05	0.02	0.02	0.85	0.42	0.42

counted

Arabidopsis lyrata	639	14	625	21.13	78.87	2.07E+008	106	2.64	0	61	0	1	35	6	3	0.08	0.03	0.03	1.28	0.61	0.61
Arabidopsis thaliana	639	9	630	23.94	76.06	1.19E+008	133	4.83	0	66	1	1	63	2	0	0.19	0.06	0.06	2.42	1.05	1.05
Oryza sativa	746	26	720	13.81	86.19	3.82E+008	59	1.59	0	9	0	1	24	17	8	0.04	0.02	0.02	0.75	0.38	0.38
Sorghum bicolor	649	70	579	7.09	92.91	7.39E+008	28	0.65	0	12	0	0	11	3	2	0.01	0.01	0.01	0.31	0.15	0.16

Pseu: tRNA pseudogenes, GS: genome size, O: Observed pairs, R: pairs of the random simulation, P p-value, Ho: Homogeneous configurations and He: Heteorgeneous configurations, ++: ->->, +-: -><-, and -+: <-->
%Cl and %noCl : percentage clustered and non clustered
