# Supplementary material for: Genomic organization of eukaryotic tRNAs
Source: BMC Genomics. 2010 Apr 28;11:270. doi: 10.1186/1471-2164-11-270 (PMC2888827; doi:10.1186/1471-2164-11-270)
Supplement: Additional file 3 — Co-occurrences of tDNAs. Comprehensive summary of co-occurrence data for tDNAs as described in Fig. 5. [file 1471-2164-11-270-S3.PDF]

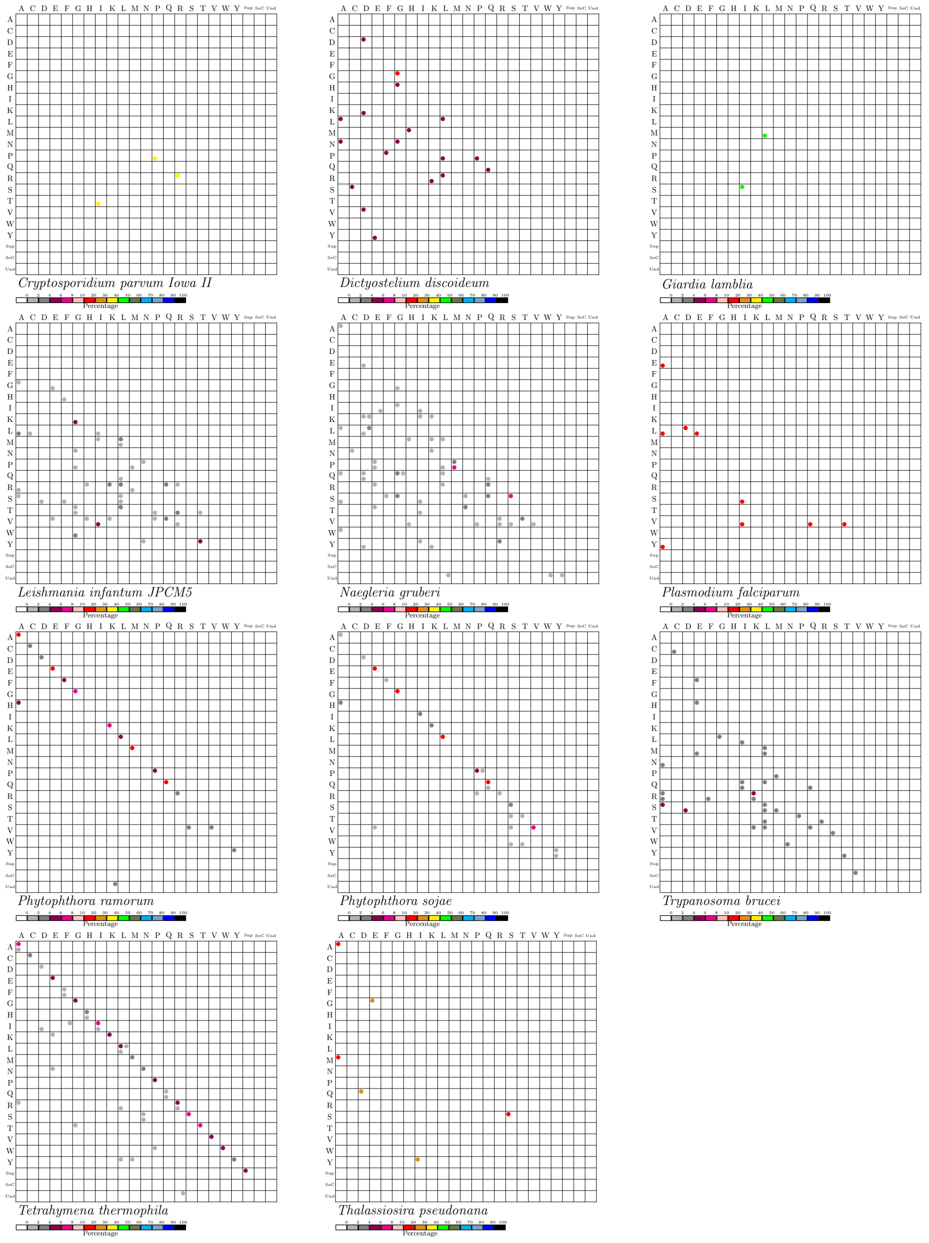

Figure 1: Protist

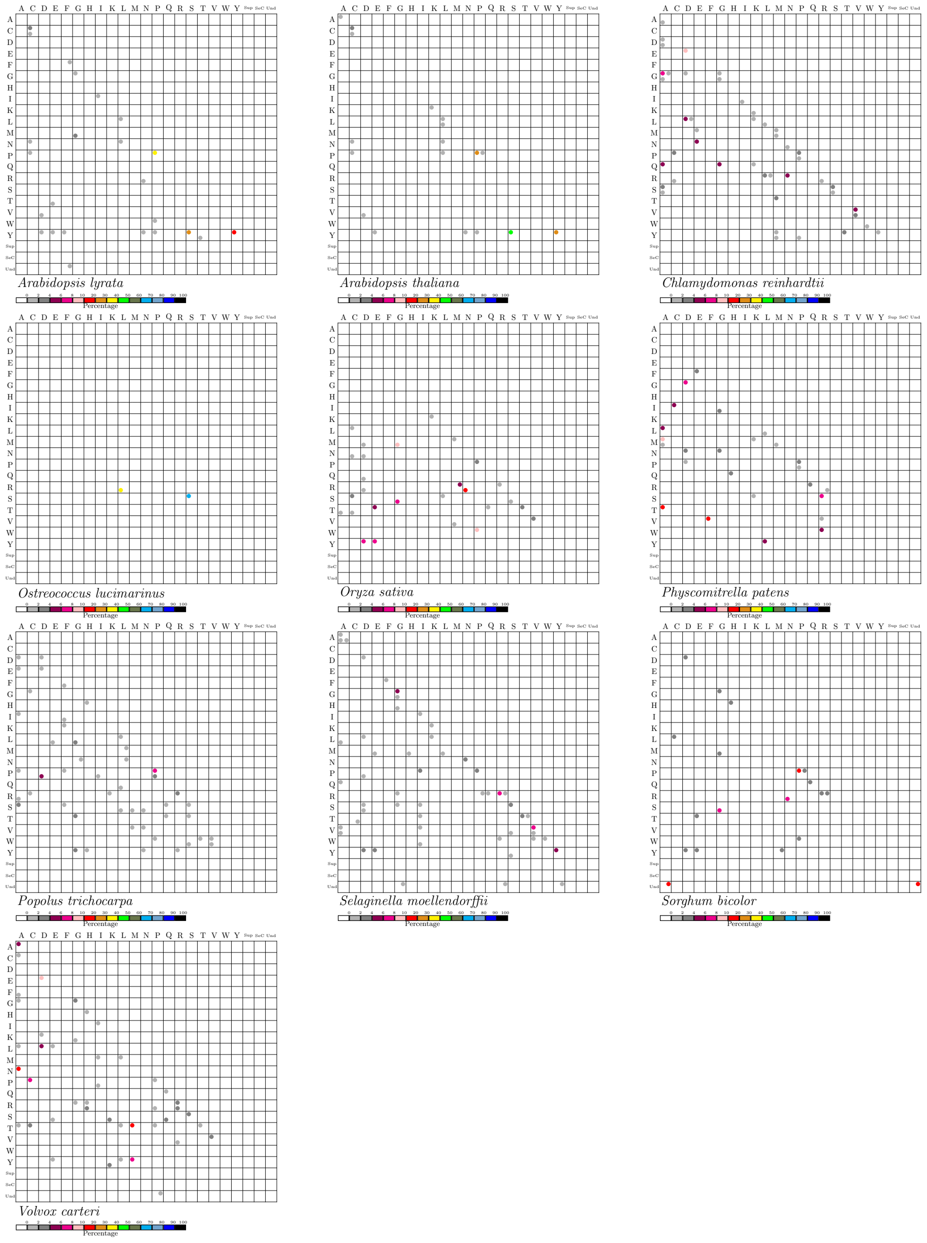

Figure 2: Green linage

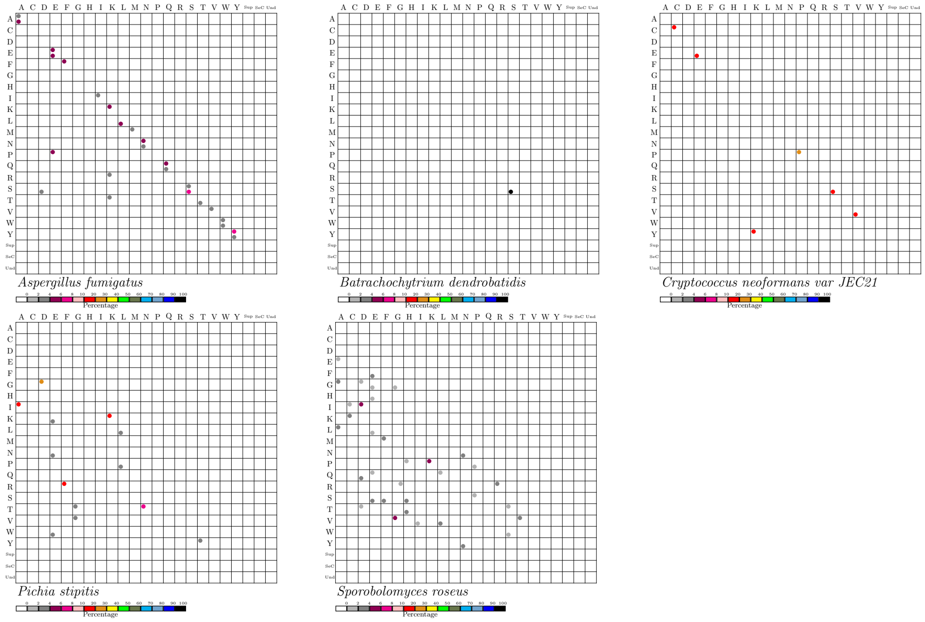

Figure 3: Fungi

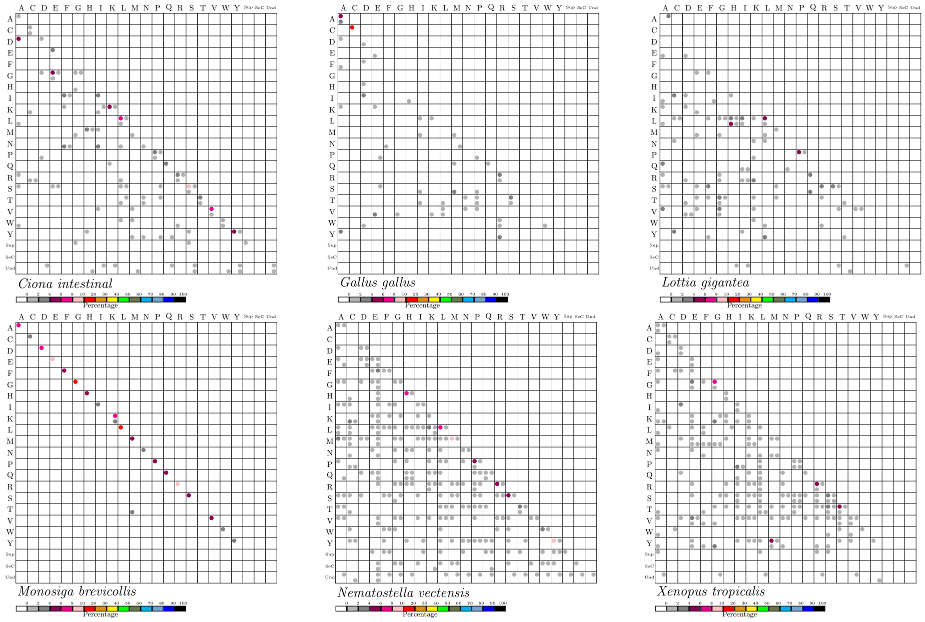

Figure 4: Non mammal animals, including the Choanoflagellate Monosiga

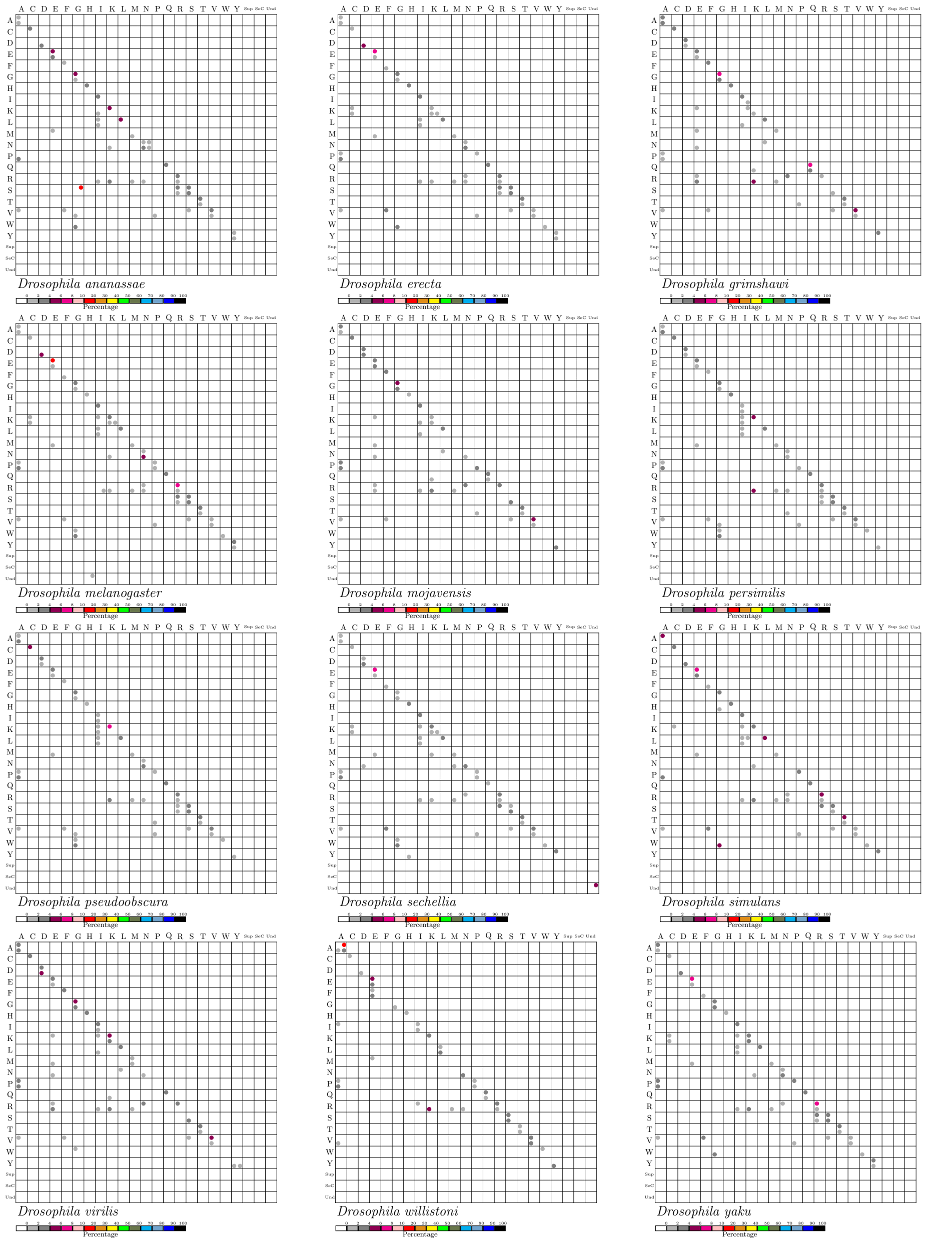

Figure 5: Drosophila genus

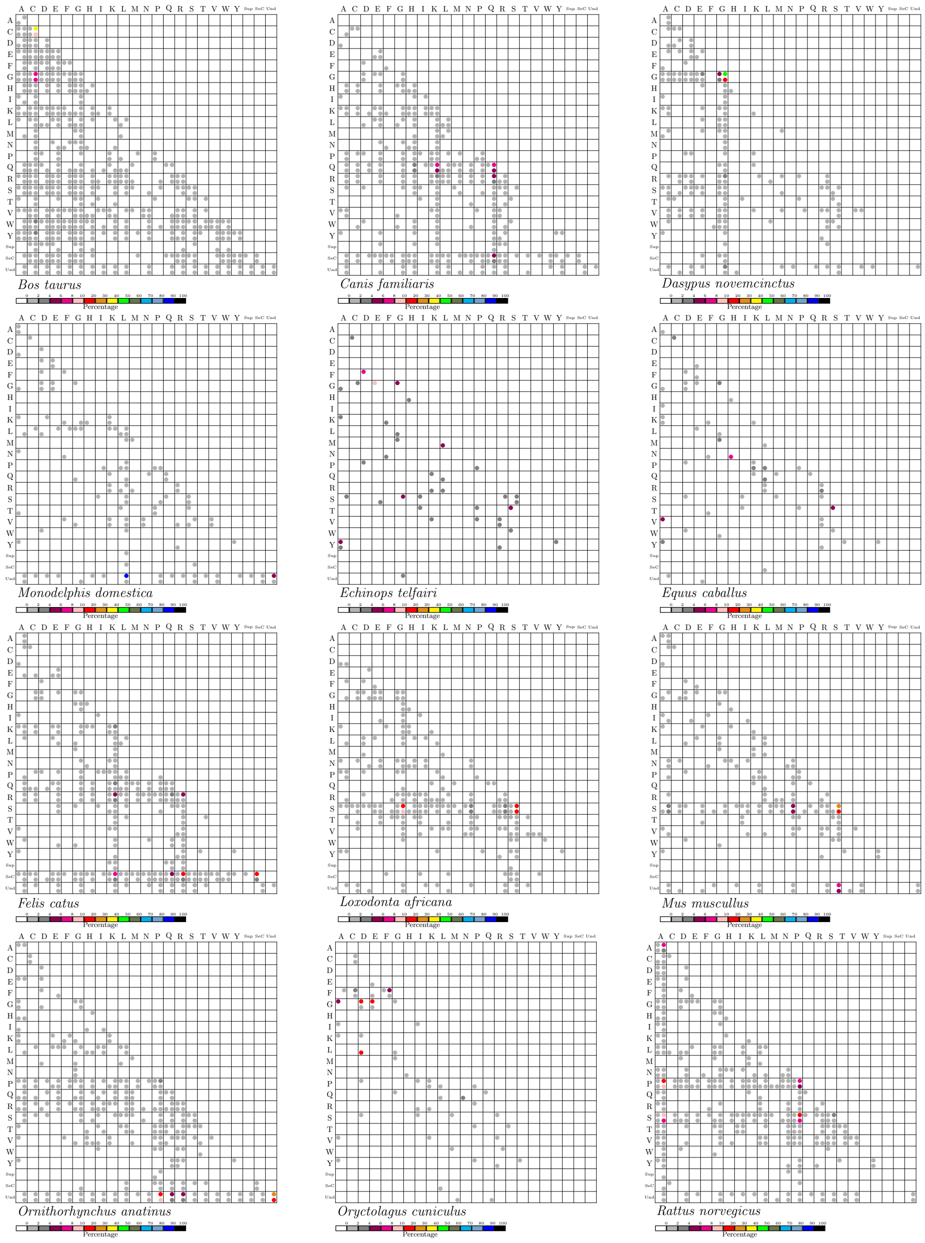

Figure 6: Non primate mammals

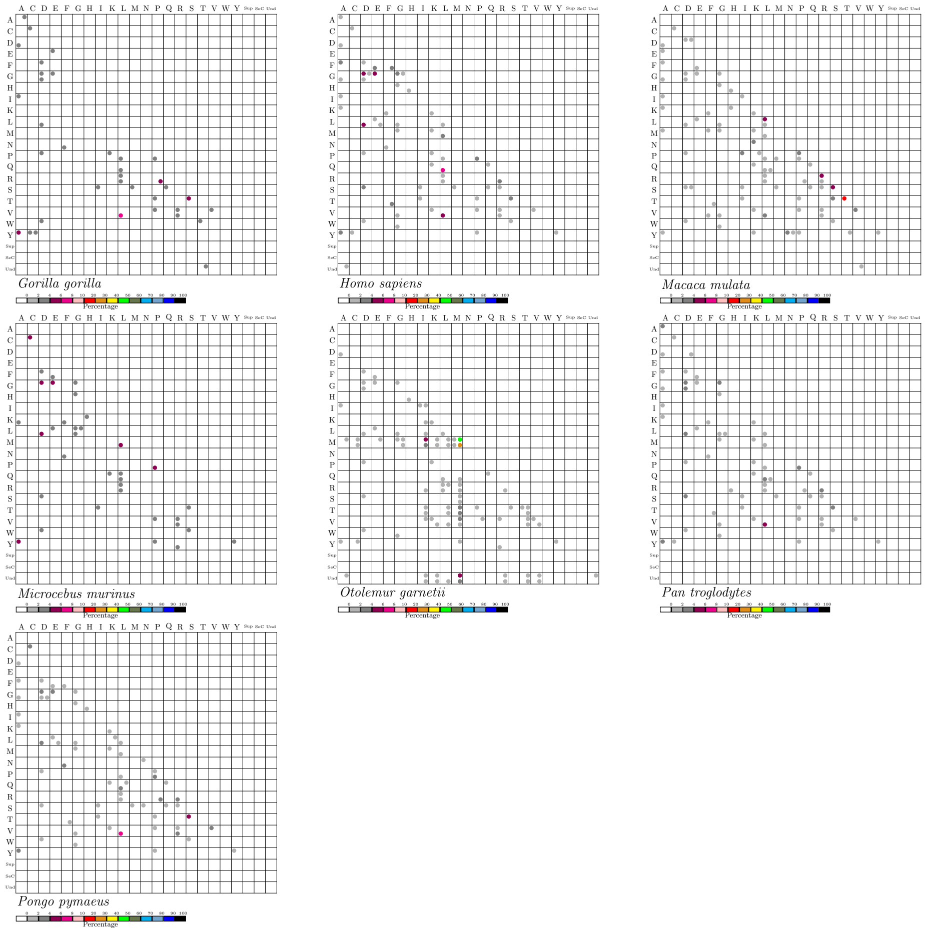

Figure 7: Primates
