# Supplementary material for: Genomic organization of eukaryotic tRNAs
Source: BMC Genomics. 2010 Apr 28;11:270. doi: 10.1186/1471-2164-11-270 (PMC2888827; doi:10.1186/1471-2164-11-270)
Supplement: Additional file 4 — Syntenic conservation of Drosophila tDNAs. List of percentage distribution of tDNA synteny in the genus Drosophila. [file 1471-2164-11-270-S4.PDF]

Additional file 3 — Synteny tables for Drosophila

Sytenic conservation of tRNAs: The table shows the percentage distribution of tRNA locis between pairs of species. Every field contains the portion of tRNAs of the species in the column, for which we could find a syntenic position in the row species.

	tRNA	Dmel	Dsec	Dsim	Dyak	Dere	Dana
**D. melanogaster**	304	-	0.78 236	0.73 222	0.64 197	0.59 180	0.54 163
**D. sechellia**	309	0.72 224	-	0.77 239	0.52 160	0.56 172	0.39 119
**D. simulans**	267	0.72 193	0.83 221	-	0.47 126	0.55 148	0.38 101
**D. yakuba**	375	0.55 207	0.43 161	0.36 134	-	0.41 152	0.42 156
**D. erecta**	284	0.67 190	0.67 190	0.61 173	0.52 149	-	0.48 137
**D. ananassae**	496	0.33 161	0.23 116	0.21 105	0.30 149	0.26 131	-
**D. pseudoobscura**	292	0.55 162	0.52 151	0.49 142	0.44 128	0.36 104	0.44 128
**D. persimilis**	298	0.49 146	0.42 126	0.38 114	0.48 142	0.43 128	0.50 150
**D. willistoni**	457	0.31 140	0.23 103	0.20 91	0.21 96	0.21 97	0.23 104
**D. mojavensis**	264	0.44 116	0.34 89	0.35 92	0.37 97	0.41 107	0.28 74
**D. virilis**	269	0.54 145	0.41 109	0.37 100	0.43 115	0.28 76	0.36 96
**D. grimshawi**	259	0.48 125	0.47 121	0.45 116	0.39 100	0.46 119	0.34 88

	tRNA	Dpse	Dper	Dwil	Dmoj	Dvir	Dgri
**D. melanogaster**	304	0.52 158	0.49 149	0.48 145	0.50 152	0.52 157	0.50 151
**D. sechellia**	309	0.43 132	0.39 122	0.31 96	0.39 122	0.37 113	0.43 133
**D. simulans**	267	0.48 129	0.37 98	0.32 85	0.43 115	0.35 94	0.45 119
**D. yakuba**	375	0.32 120	0.37 140	0.28 104	0.33 124	0.38 143	0.31 116
**D. erecta**	284	0.52 124	0.38 107	0.35 100	0.43 121	0.40 114	0.52 147
**D. ananassae**	496	0.22 109	0.30 149	0.18 88	0.21 102	0.22 111	0.20 100
**D. pseudoobscura**	292	-	0.61 178	0.32 94	0.42 123	0.41 120	0.43 125
**D. persimilis**	298	0.59 177	-	0.29 87	0.34 101	0.44 131	0.43 129
**D. willistoni**	457	0.20 91	0.17 77	-	0.18 80	0.16 74	0.18 81
**D. mojavensis**	264	0.38 101	0.31 83	0.29 76	-	0.36 94	0.45 119
**D. virilis**	269	0.41 109	0.38 103	0.29 77	0.35 95	-	0.51 138
**D. grimshawi**	259	0.39 100	0.37 96	0.30 77	0.41 107	0.51 132	-
