# Supplementary material for: Genomic organization of eukaryotic tRNAs
Source: BMC Genomics. 2010 Apr 28;11:270. doi: 10.1186/1471-2164-11-270 (PMC2888827; doi:10.1186/1471-2164-11-270)
Supplement: Additional file 5 — Sequence conservation of nematode tDNA-Ala. Neighbor-joining tree showing that tDNAs usually have identical sequences when they are syntenically conserved, while tRNAs with the same anticodon can exhibit small sequence variations within each species. [file 1471-2164-11-270-S5.PDF]

Additional file 7 — Sequence conservation of nematode tDNA-Ala.

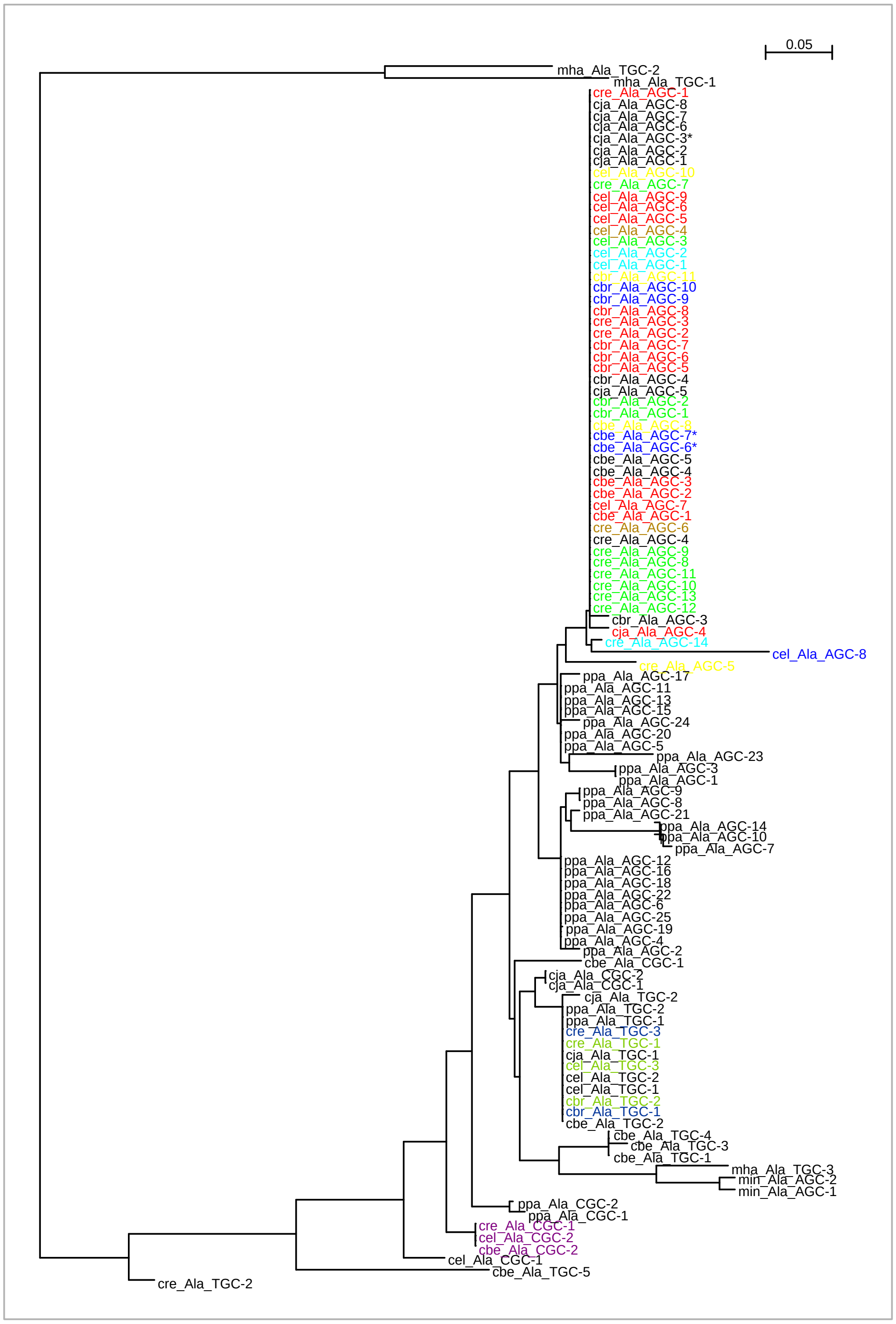

Supplemental Figure: Neighbor-joining tree of all Alanine tRNA genes in Nematodes. Each color marks a set of sequences with the same anticodon that are located in syntenic regions (see section "Synteny"). *-The sequences marked with an asterisk are additionally defined as syntenic. Most of them are identically conserved within *Caenorhabiditis*, only cel_Ala_AGC-8 (blue) has acquired several mutations and probably is in the process of losing its function. In addition to the genomes included in the survey, this figure also uses data from *Pristionchus pacificus* (ppa), *Meloidogyne hapla* (mha), and *Meloidogyne incognita* (min).
