# Supplementary material for: Genomic organization of eukaryotic tRNAs
Source: BMC Genomics. 2010 Apr 28;11:270. doi: 10.1186/1471-2164-11-270 (PMC2888827; doi:10.1186/1471-2164-11-270)
Supplement: Additional file 6 — Genome versions used in this survey. List of the genome versions and website from which they were downloaded. [file 1471-2164-11-270-S6.PDF]

S. Genome version

Code	*Specie*	Version	Source
ani	*Aspergillus_fumigatus*	Aspergillus fumigatus 2.1 01/02/08	NCBI
ary	*Arabidopsis lyrata*	Araly1	genome.jgi-psf.org
ath	*Arabidopsis thaliana*	NCBI Built 8.1	NCBI
bos	*Bos taurus*	Bos_taurus.Btau_4.0.54	ensembl.org
btr	*Batrachochytrium_dendrobatidis*	Batde5	genome.jgi-psf.org
can	*Canis_familiaris*	Canis_familiaris.BROADD2.54	ensembl.org
cbre	*Caenorhabditis_brenneri*	WS204	Wormbase
cbrig	*Caenorhabditis_briggsae*	WS204	Wormbase
cele	*Caenorhabditis_elegans*	WS204	Wormbase
chl	*Chlamydomonas_reinhardtii*	Chlre4	genome.jgi-psf.org
cio	*Ciona intestinalis*	v2.0	genome.jgi-psf.org
cjap	*Caenorhabditis_japonica*	WS204	Wormbase
cpo	*Cryptosporidium parvum Iowa II*	Cryptosporidium parvum Iowa II 07/03/2007	NCBI
crem	*Caenorhabditis_remanei*	WS204	Wormbase
cry	*Cryptococcus_neoformans_var_JEC21*	2.1 (01/07/2008)	NCBI
dan	*Danio_rerio*	Danio_rerio.Zv8.54	ensembl.org
dana	*Drosophila ananassae*	dana-all-chromosome-r1.3.fasta	FlyBase
das	*Dasypus_novemcinctus*	Dasypus_novemcinctus.dasNov2.54	ensembl.org
del	*Monodelphis_domestica*	Monodelphis_domestica.BROADO5.54	ensembl.org
dere	*Drosophila erecta*	dere-all-chromosome-r1.3.fasta	FlyBase
dgri	*Drosophila grimshawi*	dgri-all-chromosome-r1.3.fasta	FlyBase
dmel	*Drosophila melanogaster*	dmel-all-chromosome-r5.6.fasta	FlyBase
dmoj	*Drosophila mojavensis*	dmoj-all-chromosome-r1.3.fasta	FlyBase
dper	*Drosophila persimilis*	dper-all-chromosome-r1.3.fasta	FlyBase
dpse	*Drosophila pseudoobscura*	dpse-all-chromosome-r2.3.fasta	FlyBase
dsec	*Drosophila sechellia*	dsec-all-chromosome-r1.3.fasta	FlyBase
dsim	*Drosophila simulans*	dsim-all-chromosome-r1.3.fasta	FlyBase
dvir	*Drosophila virilis*	dvir-all-chromosome-r1.2.fasta	FlyBase
dwil	*Drosophila willistoni*	dwil-all-chromosome-r1.3.fasta	FlyBase
dyak	*Drosophila yakuba*	dyak-all-chromosome-r1.3.fasta	FlyBase
dyc	*Dictyostelium_discoideum*	Download march 19 2008	dictybase.org
ech	*Echinops telfairi*	Echinops_telfairi.TENREC.54	ensembl.org
equ	*Equus caballus*	Equus_caballus.EquCab2.54.	ensembl.org
fel	*Felis catus*	Felis_catus.CAT.54	ensembl.org
fug	*Takifugu rubripes*	Takifugu_rubripes.FUGU4.54	ensembl.org
gal	*Gallus gallus*	Gallus_gallus.WASHUC2.54	ensembl.org
gas	*Gasterosteus_aculeatus*	Gasterosteus_aculeatus.BROADS1.54	ensembl.org
gia	*Giardia_lamblia*	GlambliaGenomic_GiardiaDB-1.0	giardiadb.org
gor	*Gorilla gorilla*	Gorilla_gorilla.gorGor1.54	Ensembl.org
hsa	*Homo_sapiens*	NCBI36.50	ensembl.org
linf	*Leishmania infantum JPCM5*	Leishmania infantum JPCM5 30/04/2007	NCBI
lot	*Lottia gigantea*	Lotgi1	genome.jgi-psf.org
lox	*Loxodonta_africana*	Loxodonta_africana.loxAfr2.54	ensembl.org
mac	*Macaca_mulata*	Macaca_mulatta.MMUL_1.54.	Ensembl.org
mic	*Microcebus murinus*	Microcebus_murinus.micMur1.54	Ensembl.org
mon	*Monosiga_brevicollis*	Monbr1	genome.jgi-psf.org
mus	*Mus_muscullus*	Mus_musculus.NCBIM37.54	ensembl.org
naeg	*Naegleria gruberi*	Naegr1	genome.jgi-psf.org
nem	*Nematostella_vectensis*	Nemve1	genome.jgi-psf.org
oluci	*Ostreococcus lucimarinus*	release v.2.0	genome.jgi-psf.org
orn	*Ornithorhynchus_anatinus*	Ornithorhynchus_anatinus.OANA5.54	ensembl.org

ory	*Oryza_sativa*	IRGSP genome sequence build 4.0	http://rapdb.lab.nig.ac.jp/
orz	*Oryzias_latipes*	Oryzias_latipes.MEDAKA1.54	ensembl.org
oto	*Otolemur_garnettii*	Otolemur_garnettii.BUSHBABY1.54	ensembl.org
pan	*Pan troglodytes*	Pan_troglodytes.CHIMP2.1.54	Ensembl.org
pfal	*Plasmodium_falciparum*	Plasmodium falciparum 3D7 (14-11-2007)	NCBI
phy	*Physcomitrella_patens*	P. patens subsp patens v1.1	genome.jgi-psf.org
pic	*Pichia stipitis*	Pichia stipitis v2	genome.jgi-psf.org
pon	*Pongo_pygmaeus*	Pongo_pygmaeus.PPYG2.54	ensembl.org
pop	*Popolus_trichocarpa*	Populus trichocarpa v1.1	genome.jgi-psf.org
pram	*Phytophthora ramorum*	Phyra1_1	genome.jgi-psf.org
psoj	*Phytophthora sojae*	Physo1_1	genome.jgi-psf.org
rab	*Oryctolagus_cuniculus*	Oryctolagus_cuniculus.RABBIT.54	ensembl.org
rat	*Rattus norvegicus*	Rattus_norvegicus.RGSC3.4.54	Ensembl.org
sel	*Selaginella_moellendorffii*	Selmo1	genome.jgi-psf.org
sor	*Sorghum_bicolor*	Sorbi1	genome.jgi-psf.org
spo	*Sporobolomyces_roseus*	Sporo1	genome.jgi-psf.org
tbru	*Trypanosoma_brucei*	Trypanosoma brucei TREU927 (30-10-2006)	NCBI
tet	*Tetrahymena_thermophila*	Assembly2--Nov_2003	www.tigr.org
thala	*Thalassiosira_pseudonana*	Thaps3	genome.jgi-psf.org
tpax	*Trichoplax_adhaerans*	Triad1	genome.jgi-psf.org
tra	*Tetraodon_nigroviridis*	Tetraodon_nigroviridis.TETRAODON8.54	Ensembl.org
vol	*Volvox_carteri*	Volca1	genome.jgi-psf.org
xen	*Xenopus_tropicalis*	Xenopus_tropicalis.JGI4.1.54	ensembl.org
