# Supplementary material for: Genomic organization of eukaryotic tRNAs
Source: BMC Genomics. 2010 Apr 28;11:270. doi: 10.1186/1471-2164-11-270 (PMC2888827; doi:10.1186/1471-2164-11-270)
Supplement: Additional file 7 — Proportions of tDNA pairs. Summary of the Fisher Test statistic for comparing proportion of pairs configurations. [file 1471-2164-11-270-S7.PDF]

S. Proportion comparisons of raw pair counts and filtered pair counts after removing tRNA pseudogenes

Specie	Homogenous pair raw	Homogenous pair filtered	Heterogenous pair raw	Heterogenous pair filtered	P value	ODDS RATIO
Trypanosoma brucei	2	2	35	35	1	1
Leishmania infantum	2	2	56	56	1	1
Naegleria gruberi	12	12	65	59	1	0.91
Giardia lamblia	0	0	2	2	No determinated	
Plasmodium falciparum	0	0	9	9	No determinated	
Cryptosporidium parvum	2	2	1	1	1	1
Tetrahymena thermophila	321	322	20	16	0.61	0.8
Phytophthora sojae	58	58	9	8	1	0.89
Phytophthora ramorum	34	34	4	4	1	1
Thalassiosira pseudonana	2	2	7	7	1	1
Dictyostelium discoideum	6	6	13	13	1	1
Batrachochytrium dendrobatidis	1	1	0	0	No determinated	
Sporobolomyces roseus	6	6	50	50	1	1
Cryptococcus neoformans	6	6	1	1	1	1
Aspergillus fumigatus	33	33	5	5	1	1
Pichia stipitis	5	5	25	25	1	1
Monosiga brevicollis	47	47	2	1	1	0.5
Trichoplax adhaerans	0	0	0	0	No determinated	
Nematostella vectensis	8776	9782	5367	3810	3.86E-069	0.64
Lottia gigantea	104	105	525	510	0.82	0.96
Drosophila simulans	55	55	28	27	1	0.96
Drosophila sechellia	78	78	35	34	1	0.97
Drosophila melanogaster	91	91	33	30	0.77	0.91
Drosophila yakuba	93	93	36	35	1	0.97
Drosophila erecta	78	78	32	31	1	0.97
Drosophila ananassae	89	89	52	52	1	1
Drosophila pseudoobscura	76	76	31	31	1	1
Drosophila persimilis	73	73	34	34	1	1
Drosophila willistoni	84	84	25	25	1	1
Drosophila mojavensis	55	55	24	24	1	1
Drosophila virilis	60	60	26	25	1	0.96
Drosophila grimshawi	52	52	24	23	1	0.96

Caenorhabditis briggsae	105	105	67	55	0.43	0.82
Caenorhabditis remanei	76	76	58	50	0.61	0.86
Caenorhabditis brenneri	130	131	103	84	0.29	0.81
Caenorhabditis elegans	127	127	68	63	0.75	0.93
Caenorhabditis japonica	97	97	54	41	0.32	0.76
Ciona intestinalis	290	302	250	207	0.07	0.8
Danio rerio	5124	5895	9543	7743	2.40E-046	0.71
Tetraodon nigroviridis	66	66	51	40	0.41	0.79
Takifugu rubripes	93	94	38	29	0.39	0.76
Gasterosteus aculeatus	1987	2033	1157	1027	0.01	0.87
Oryzias latipes	166	169	120	96	0.19	0.79
Xenopus tropicalis	590	597	1178	1109	0.32	0.93
Ornithorhynchus anatinus	12107	12134	14908	14048	0	0.94
Monodelphis domestica	156	156	7246	7148	0.91	0.99
Dasypus novemcinctus	2381	2398	5537	2353	1.90E-115	0.42
Oryctolagus cuniculus	16	16	102	99	1	0.97
Mus muscullus	403	403	598	449	0	0.75
Rattus norvegicus	5463	5629	22735	16009	2.35E-069	0.68
Echinops telfairi	6	6	43	38	1	0.88
Canis familiaris	761	769	4097	2614	8.08E-016	0.63
Felis catus	2084	2138	6708	2398	3.13E-163	0.35
Bos taurus	7724	7822	20728	13660	4.99E-108	0.65
Equus caballus	14	14	58	57	1	0.98
Loxodonta africana	359	363	1286	712	9.30E-012	0.55
Otolemur garnettii	826	826	538	430	0.01	0.8
Microcebus murinus	6	6	36	35	1	0.97
Macaca mulata	73	73	95	87	0.74	0.92
Pongo pygmaeus	17	17	66	61	0.85	0.92
Gorilla gorilla	4	4	36	31	1	0.86
Pan troglodytes	16	16	62	58	1	0.94
Homo sapiens	15	15	82	75	0.84	0.92
Gallus gallus	22	22	44	42	1	0.95
Ostreococcus lucimarinus	2	2	1	1	1	1
Chlamydomonas reinhardtii	35	35	101	99	1	0.98

Volvox carteri	54	54	195	194	1	0.99
Physcomitrella patens	6	6	79	78	1	0.99
Selaginella moellendorffii	52	52	58	52	0.78	0.9
Popolus trichocarpa	13	13	46	44	1	0.96
Arabidopsis lyrata	62	62	44	44	1	1
Arabidopsis thaliana	68	68	65	64	1	0.98
Oryza sativa	10	10	49	46	1	0.94
Sorghum bicolor	12	13	16	10	0.4	0.58
